# Self-evaluation and evaluation of nursing leaders Leadership Styles*

**DOI:** 10.1590/1518-8345.3435.3393

**Published:** 2021-05-21

**Authors:** Angie Lorena Riao Castillo, Ma. Elsa Rodrguez Padilla, Daro Gaytn Hernndez

**Affiliations:** 1 Universidad Autnoma de San Luis Potos, Facultad de Enfermera y Nutricin, San Luis Potos, San Luis Potos, Mexico.

**Keywords:** Self-Assessment, Evaluation, Leadership, Nursing, Nursing, Supervisory, Workforce, Autoavaliao, Avaliao, Liderana, Enfermagem, Superviso de Enfermagem, Recursos Humanos, Autoevaluacin, Evaluacin, Liderazgo, Enfermera, Supervisin de Enfermera, Recursos Humanos

## Abstract

**Objective:**

to evaluate the concordance between the leadership styles self-evaluated by the Nursing managers and evaluated by their subordinates in a private hospital.

**Methodology:**

an observational, cross-sectional, quantitative, and analytical study, with population of 31 managing nurses and 125 subordinates. Herman Bachenheimers instrument of Situational Leadership was employed, adapting it to the subordinates. The concordance between self-evaluation and evaluation by the subordinates was analyzed in the four leadership styles (Directing, Guiding, Participating, Delegating), with the Kappa coefficient statistical test, test statistic (Z) >1.96, 95% confidence interval and PASW Statistics, version 18.

**Results:**

the self-evaluation of the Nursing managerial staff has a tendency for the Guiding Style and, according to the evaluation by their subordinates, there is a minimum difference among the four styles. Their concordance is low, but significant, with 19.3%. It was identified that the subordinates perceive that they possess the necessary competences to autonomously perform the tasks assigned, and that there is trust and assertive communication between both groups, which facilitates knowledge exchange.

**Conclusion:**

the Nursing managers and their subordinates perceive various leadership styles, and concordance is low. To attain superior leadership styles, the subordinates must develop autonomy and empowerment.

## Abstract (pt)

**Objetivo:**

avaliar a concordncia entre os estilos de liderana autoavaliados pelos gerentes de enfermagem e avaliados pelos prprios liderados em um hospital privado.

**Mtodo:**

estudo observacional, transversal, quantitativo e analtico, com populao de 31 enfermeiras gestoras e 125 liderados. Foi utilizado o instrumento de Liderana Situacional de Herman Bachenheimer, adaptando-o para os liderados. Foi analisada a concordncia entre a autoavaliao e a avaliao dos liderados nos quatro estilos de liderana (Determinar, Persuadir, Compartilhar, Delegar), com o teste de significncia estatstica coeficiente Kappa, teste estatstico (Z)> 1,96, intervalo de confiana de 95 % e programa *PASW Statistics* verso 18.

**Resultados:**

a autoavaliao dos gerentes de enfermagem tende ao estilo Persuadir e, de acordo com a avaliao de seus liderados, existe diferena mnima entre os quatro estilos. A concordncia pobre, porm significativa com 19,3%. Identificou-se que os liderados consideram-se a si mesmos como possuidores das competncias necessrias para realizar as tarefas atribudas com autonomia, h confiana e comunicao assertiva entre ambos os grupos, o que facilita a troca de conhecimentos.

**Concluso:**

os gerentes de enfermagem e seus liderados reconhecem diferentes estilos de liderana e a concordncia pobre. Para alcanar estilos de liderana superiores, os liderados devem desenvolver autonomia e empoderamento.

## Abstract (es)

**Objetivo:**

evaluar la concordancia entre los estilos de liderazgo autoevaluados por los directivos de enfermera y evaluados por sus propios seguidores en un hospital privado.

**Mtodo:**

estudio observacional, transversal, cuantitativo y analtico con poblacin de 31 enfermeras directivas y 125 seguidores. Se emple el instrumento de Liderazgo Situacional de Herman Bachenheimer, adaptndolo para los seguidores. Se analiz la concordancia entre la autoevaluacin y evaluacin de los seguidores en los cuatro estilos de liderazgo (Dirigir, Guiar, Participar, Delegar), con la prueba estadstica coeficiente de Kappa, estadstico de prueba (Z) >1,96, intervalo de confianza del 95% y programa *PASW Statistics* versin 18.

**Resultados:**

la autoevaluacin del personal directivo de enfermera tiende al estilo Guiar y, segn la evaluacin por sus seguidores, hay mnima diferencia entre los cuatro estilos. Su concordancia es pobre, pero significativa con el 19,3%. Se identific que los seguidores se perciben con las competencias necesarias para realizar con autonoma las tareas asignadas, existe confianza y comunicacin asertiva entre ambos grupos, lo que facilita intercambio de conocimientos.

**Conclusin:**

los directivos de enfermera y sus seguidores perciben diversos estilos de liderazgo, y la concordancia es pobre. Para alcanzar estilos de liderazgo superiores, los seguidores deben desarrollar autonoma y empoderamiento.

## Introduction

It is but a challenge to keep professionals with sufficient competences to occupy leadership roles, since the Nursing professionals who hold Leadership roles must be able to influence on the mechanisms for the adoption of decisions that establish priorities, as well as to allocate resources to attain health^(^ 1 ^)^; likewise, the lack of managerial and leadership ability at all levels of the health system is more frequently cited as a determinant obstacle to improving care quality, expanding the health services, and attaining the development goals of the millennium^(^ 2 ^-^ 3 ^)^.

On the other hand, to achieve the 2030 Sustainable Development Objectives and to address a whole series of challenges, including the worldwide scarcity of Nursing staff, it is necessary that the national Nursing leaders of the world work together to formulate a strategy for long-term sustainable development, promoting the progress of the Nursing science, the development of the Nursing course, the advance of industrial Nursing, and the improvement of peoples general health^(^ 4 ^)^.

It is also acknowledged that the Nursing leaders are integral actors not only in the provision of quality medical care, but also in operational excellence in various medical care environments^(^ 5 ^)^; in addition, it is considered that leadership plays a fundamental role in the nurses lives and requires strong, coherent, and well-informed leaders^(^ 1 ^)^.

Therefore, well-prepared leaders are required who are able to assume the corresponding role, influence on their subordinates (the individuals who are responsible for the direct care provided to the patients), and favor conducts and behaviors in the health staff; these conditions are fundamental so that the Nursing practice can advance to a leadership with a favorable and participative behavior which generates a working atmosphere that eases teamwork with good communication, respect and team autonomy, thus actively including their personnel in the decisions, for a humanized and good quality management practice^(^ 1 ^)^.

The Situational Leadership model proposed by Hersey & Kenneth H. Blanchard, identified as the Situational Leadership Theory (SLT), emerges from the basic principle that the leaders must adapt to the situation prevailing in the organization, that is to say, if the situation changes, the leaders must be able to change and adapt to this new situation in order to attain the goals and objectives that have been set out; their efficiency for their subordinates to be well-directed and to identify them as a guide who generates trust will depend on that. This leadership style is defined as the process of influencing on the activities of an individual or group in the efforts to attain goals in a given situation, attributing special relevance to the situation in which each leader can have a preferred style^(^ 6 ^)^.

This model implies the integration of two dimensions: task or relationship conduct (similar to those defined by the University of Ohio), according to the different situations experienced. The first refers to the communication from the leaders to their subordinates in a detailed manner regarding the tasks to be performed, indicating specifications that leave no room for doubts; the second refers to bilateral communication, in order to offer support not only in relation to the assigned task but also to situations relating to personal emotions, health, and communication among peers, since the leaders listen, guide, and support their subordinates^(^ 3 ^)^.

According to these two behavioral dimensions, the leaders have two possibilities (high or low) in the four leadership styles (Directing, Guiding, Participating, Delegating); in the first, they monitor, give specific instructions, and closely supervise performance, are little encouraging and very governing; in the second, they explain to their subordinates aspects referring to the decisions made and allow them to clarify situations; the third style is characterized by sharing ideas, making suggestions, and conveying confidence to the subordinates so that they take risks and, in the fourth, they transfer responsibility for the decisions and their materialization, there is autonomy and trust, and the subordinates are duly qualified and trained^(^ 7 ^)^.

In this style, the behavior that the leaders adopt with their subordinates regarding the tasks is of paramount importance, since an harmonic and dynamic work environment where interaction is enabled both among the subordinates and between subordinates and leaders is required by public and private institutions, integrating professional nursing staff with sufficient competences, efficient and prepared to act as leaders, capable of managing adequately with reliability and innovation^(^ 1 ^)^, in addition to being able to successfully face the changes required by the situation; to such end, they must have the skill and ability to modify their style as necessary^(^ 8 ^)^.

The leaders have indicated that it is difficult to solve conflicts and to carry out their leadership activities^(^ 1 ^)^, there is a tendency in the leaders to perform tasks instead of having their subordinates perform them^(^ 9 ^)^, and the main activities are those regarding the accomplishment of the task^(^ 7 ^)^, leaving to a second place the relationships with the subordinates, which becomes more noticeable with the high workloads which are the result, among other causes, of the demands from the institutions, the need to meet the established goals, and the diversity of characteristics in terms of aptitudes, attitudes, and capabilities of the personnel under their charge^(^ 1 ^)^.

There is not enough data referring to the evaluations conducted on the Nursing managers by their subordinates, although the results of a research study show that only three of the four learning styles are frequently used, which, according to their frequency of use and in descending order, are the following: Delegating, Guiding, and Directing^(^ 10 ^)^. It is believed that this situation can be related to the managers control scope, which has undergone an expansion due to the reduction in the number of intermediate managers in their organizational structure, thus extending the control space^(^ 10 ^)^.

However, society does not value the leadership process^(^ 1 ^)^; on the other hand, assuming leadership is no guarantee as to its efficacy^(^ 11 ^)^ but, in addition to influencing on the direct care provided to the patients, it also exerts an influence on other important aspects like administration, education, decision-making, and peer autonomy, among others^(^ 1 ^)^. In view of the aforementioned, for Nursing leaders to provide quality care, they must possess certain characteristics, such as the competences which allow for teamwork^(^ 2 ^)^, combined with the need to analyze the current preparation of the nursing professionals to exercise leadership; but not only that, they must also wonder if they are seen as prepared to adapt to different leadership styles according to the situation and to verify if there is concordance between their self-evaluations and the evaluations by their subordinates.

One of the theories that can contribute for the Nursing leaders to visualize different perspectives and, in doing so, favor their own professional development and that of their subordinates, as well as the managerial skills, is Joharis window; according to this theory, it is recommended that the best area where Nursing managerial staff can be found is the free area, since it allows exchanging information among peers and subordinates, thus favoring interpersonal relationships, among other beneficial aspects to attain the institutions goals and objectives^(^ 12 ^)^.

It is for this reason that, when solving those questions in future studies, it could direct the strengthening of the quantitative trend on leadership and its styles according to the SLT, as well as it would bolster the theoretical and empirical knowledge of the existing concordance between the leader, the subordinate, and Joharis window, which could go beyond what some authors have expressed directed to the relation with negative feelings^(^ 13 ^)^, proactive personality^(^ 14 ^)^, satisfaction^(^ 15 ^)^, innovation leadership predictors, and self-esteem in the subordinates^(^ 16 ^)^.

The purpose of this study was to evaluate the concordance between the leadership styles self-evaluated by the nursing managers and evaluated by their own subordinates in a private hospital.

## Method

This is an observational, cross-sectional, quantitative, and analytical study, conducted in a private hospital in the city of San Luis Potos, Mexico. The target population was made up by 31 managing nurses and 125 of their subordinates; in turn, 30 of these leaders participated as subordinates.

The criteria for inclusion considered were the following: leaders with a managerial position, with no age limit, with a minimum of 6 months in their current positions; regarding the subordinates, they had to have a leader at the managerial level, with no age limit, and a minimum of 1 month working with the leader; both had to accept to participate and voluntarily sign the informed consent. Finally, the exclusion criteria contemplated were the following: leaders and subordinates who were on vacation, on disability leave, or those who have not participated voluntarily in the research.

The project was approved by the Ethics Committee of the Nursing and Nutrition School (CEIFE-2016-185) and by the Ethics and Research Committee of the private hospital. The ethical considerations set forth in the regulations of the General Law on Health Regarding Research in Health of the Mexican United States were met, as well as the Declaration of Helsinki by the World Medical Association for medical research studies, and the inclusion of the participants who read and signed the informed consent letter, freely and independently.

Two structured instruments were used for data collection, enumerated, without disclosing the name of the manager evaluated by the respective subordinates and without any kind of personal data of the evaluators; each survey lasted approximately 30 minutes and the managers and subordinates freedom to withdraw from the study at any moment was respected at all times. Data collection was carried out from January to June 2017.

The dependent variables analyzed were the four situational leadership styles described in Hersey & Blanchards theory, namely: Directing, Guiding, Participating, and Delegating. The independent variables were age, gender, job position, time working in the position, and time working as a leader.

For data collection, an instrument by Riao and Rodrguez that assesses situational leadership was adapted to the Mexican population; an item was added with sociodemographic and work data. The instrument has 17 questions with four answer items each instrument, indicating the four situational leadership styles with content and construct validation, both for the leaders self-evaluation and for the evaluation by the subordinates. This instrument had been previously used in a study called Situational leadership in nurses working in a health institution of Bucaramanga (Colombia)^(^ 17 ^)^. It was originally designed by Dr. Herman Bachenheimer, who conducted the entire validation process.

The usual situation in any organization is that the leaders and their subordinates indicate the actions that the leaders must take, that is, the choice of their behavior when facing these situations; in this way, an analysis is accomplished of the different leadership styles and of their adaptability to the new needs, so that it is possible to identify their flexibility and ability to change their style.

To identify the leadership style, the answers which fit each style were considered; based on the transformation table attached to the instrument, the leadership style with the highest total percentage was considered. The possible answers were labeled as A, B, C, and D, without any pre-established order, or with different values; each column referred to a different style.

Data collection was conducted in three stages: in the first, the instrument was applied to the managerial staff (Nurse in Chief, Nursing Supervisors, Service Chiefs, and Shift Managers) and to their corresponding subordinates; in the second, the instrument was applied to the leaders to evaluate the manager and, in the third, the second instrument seen by the subordinates was applied to evaluate the leader who has to be assessed by the subordinate. In all the cases, the applications were responsibility of the lead researcher and, before applying the instrument, the participants were given the informed consent form; the instrument was applied after they signed such form.

Data capture was performed in Excel and, for their processing, they were exported to PASW Statistics, version 18, in Spanish. Relative and absolute frequencies were estimated in the qualitative variables and, for the quantitative ones, some measures of central tendency and dispersion were estimated. To evaluate the concordance between the leaders self-evaluation and the evaluation by the subordinates with respect to the four leadership styles (Directing, Guiding, Participating, and Delegating), the Kappa coefficient statistical test was used, observing that the test statistic (Z) was higher than 1.96; in order to consider a significant concordance, such criterion is similar to p<0.05, when working with a 95% confidence interval.

## Results

Table 1 shows the results of the self-evaluation of the Nursing managerial staff, and the variations in the results are identified; globally, the styles with the highest and lowest percentages were Guiding and Delegating, respectively; when analyzing by job position: the Nurse in Chief was identified as a leader with the Directing style, and the Nursing Supervisors, Service Chiefs, and Shift Managers are visualized as employing the Guiding style: both globally and by job position, the Delegating style was the least identified.

**Table 1:** Self-evaluation of the situational leadership styles by the Nursing managerial staff (n=31) of a private hospital, by job position and in a global manner. San Luis Potos, S.L.P., Mexico, 2017

	Directing		Guiding		Participating		Delegating	
	N	%	n	%	n	%	n	%
Nurse in Chief	8	47.1	7	41.2	2	11.8	0	0.0
Nursing Supervisors	33	32.4	34	33.3	29	28.4	6	5.9
Service Chiefs	35	25.7	55	40.4	40	29.4	6	4.4
Shift Managers	52	20.4	95	37.3	87	34.1	21	8.2
*Global Result*	*128*	*25.1*	*191*	*37.5*	*158*	*31.0*	*33*	*6.5*

According to Table 2, the leadership style is very variable: the subordinates identify that, globally, the leaders apply the Guiding style first and that Directing is the one they practice the least. Likewise, the Nurse in Chief mainly makes use of Participating, the Nursing Supervisors of Directing, the Service Chiefs of Delegating, and the Shift Managers of Participating. It is important to clearly identify that only the Service Chiefs delegate and that both the Nurse in Chief and the Shift Managers Participate and Guide, whereas the Nursing Supervisors direct and Guide.

**Table 2:** Evaluation of the situational leadership styles by the subordinates of the Nursing managerial staff of a private hospital, by job position and in a global manner. San Luis Potos, S.L.P., Mexico, 2017

	n=125							
	Directing		Guiding		Participating		Delegating	
	N	%	n	%	n	%	n	%
Nurse in Chief	32	26.9	33	27.7	34	28.6	20	16.8
Nursing Supervisors	162	27.2	156	26.2	138	23.2	139	23.4
Service Chiefs	138	20.3	181	26.6	168	24.7	193	28.4
Shift Managers	173	22.6	213	27.8	219	28.6	160	20.9
*Global Result*	*505*	*23.4*	*583*	*27.0*	*559*	*25.9*	*512*	*23.7*

Table 3 shows the concordance between the leaders self-evaluation and the evaluation by the subordinates; only in the case of the Nurse in Chief no significant concordance was found (Z=-0.656); whereas with the Nursing Supervisors, the Service Chiefs, and the Shift Managers there was low concordance, but significant (Z>1.96), while good and significant concordance was found globally.

**Table 3:** Concordance between the self-evaluation and the evaluation of the situational leadership styles by the subordinates of the Nursing managerial staff, by job position and in a global manner San Luis Potos, S.L.P., Mexico, 2017

n=125		
Position	K*	Z^[Table-fn TFN2]^
**Nurse in Chief**	**-0.037**	**-0.656**
**Nursing Supervisors**	**0.119**	**4.817**
**Service Chiefs**	**0.058**	**2.657**
**Shift Managers**	**0.090**	**4.085**
*Global*	*0.82*	*6.426*

* Kappa Coefficient;

Test value (1.96)

Table 4 shows that, in the vast majority (80.65%) of the leaders, their self-evaluation did not coincide with that of their subordinates. According to the job position, the Nurse in Chief recorded concordance in 0.0%; the Nursing Supervisors in 50.0%; the Service Chiefs in 12.5% and Shift Managers in 12.5%; and there was global concordance in 19.35%.

**Table 4:** Concordance between the self-evaluation and the evaluation of the situational leadership styles by the subordinates, by each Nursing Manager of a private hospital. San Luis Potos, S.L.P., Mexico, 2017

n=31			
Position	Leader	K*	Z^[Table-fn TFN4]^
Nurse in Chief	1	-0.037	-0.656
Nursing Supervisors	2	-0.028	-0.196
	3	0.264	1.540
	4	0.120	2.140
	5	0.196	3.525
	6	0.115	2.423
	7	0.036	0.785
Service Chiefs	8	0.067	1.791
	9	0.134	0.952
	10	0.098	0.847
	11	-0.035	-0.490
	12	0.180	2.083
	13	0.006	0.080
	14	-0.003	-0.080
	15	0.094	1.088
Shift Managers	16	0.011	0.250
	17	0.183	3.111
	18	0.065	1.122
	19	0.410	2.066
	20	0.080	0.932
	21	0.042	0.412
	22	0.141	1.216
	23	0.162	1.581
	24	0.048	0.623
	25	0.214	1.959
	26	-0.128	-1.002
	27	0.257	1.671
	28	0.165	1.925
	29	0.173	1.580
	30	0.032	0.330
	31	-0.030	-0.184

* Kappa Coefficient;

Test value (1.96)

## Discussion

The Nursing managerial staff presents different leadership styles, within the framework of the two conducts established in the model itself, with the Guiding Style showing significance from the managers self-evaluation, followed by Participating, Directing, and Delegating. These findings were consistent with those of a study conducted in Mexico^(^ 18 ^)^ and with studies from Colombia^(^ 7 ^)^ and Spain^(^ 19 ^)^, a fact that signals that there has not been any change for an extended period of time. The Participating style prevails in second place, with Delegating last. However, there is a study conducted in Chile which does not coincide with the findings of this research, with its starting point based on the fact that there is no leadership style better than other, but more adequate for each situation. The style they mostly perceive is Guiding, followed by Delegating, Participating and, to a lesser extent, Directing^(^ 3 ^)^.

Now, based on the analysis by job position, the Nurse in Chief and the Nursing Supervisors employ a style towards Guiding, task-oriented, which might be related to the political-institutional demands and to the mandatory compliance goals which, according to a study conducted in Chile, leave aside the motivating and encouraging conducts for the staff to attain outstanding performance^(^ 1 ^)^.

Likewise, a study conducted in Pakistan and another from Brazil suggest that, to attain higher leadership styles, the leaders must improve their personal relationships, with particular interest in their subordinates needs^(^ 20 ^-^ 21 ^)^, as well as show concern for the personal development of each subordinate^(^ 1 ^)^. The participative style focuses on communication and on the relationships with the subordinates so as to achieve better results in health and to overcome the challenges inherent to the profession^(^ 3 ^,^ 7 ^,^ 20 ^-^ 21 ^)^; and, for the delegative style, this is only possible when the subordinates possess a high level of preparation and when they are sufficiently motivated to perform the task, since the leader includes them in a task in a more direct manner^(^ 22 ^)^ and knows that they have the necessary skills and knowledge to perform it^(^ 7 ^)^.

Therefore, the leaders are agents for transformation and need to work hard with their subordinates training them in their job positions, so that they can perform autonomously, solve conflicts, and make decisions^(^ 22 ^-^ 24 ^)^, thus managing to empower them in the assigned and delegated activities^(^ 9 ^,^ 23 ^-^ 24 ^)^. The aforementioned would promote the subordinates to be labeled as leaders^(^ 25 ^)^ of their own process in providing direct quality care. However, it is essential that the institutional policies, as well as the managers of each institution, present a transformational view to attain the high leadership styles and outstanding performance in the achievement of the objectives set out^(^ 1 ^)^.

However, the subordinates identify that their leaders employ the four leadership styles with a minimum difference among them, which means that the leaders do not have a single and preferred style, that they act differently according to the circumstances that the institution is going through, and that their leadership is molded according to the ongoing situation, as the SLT states. However, there is greater predominance of the Guiding style, followed by Participating, Delegating, and Directing.

Likewise, the subordinates perceive a supporting behavior and bilateral communication, where they can participate autonomously in decision-making and in conflict resolution, thus being conducts guided towards participation and delegation by the Nurse in Chief, the Service Chiefs, and the Shift Managers.

However, these results that have been detected differ from others published, where the subordinates report needing a Guiding leadership style (high in direction and in support), but that the most frequent leadership style they receive is Delegating, followed by Guiding and Participating (33%), and by Directing (3%). That is to say, 42% reported the Delegating leadership style (low in direction and in communication), and only 13% mentioned needing it. The possible explanation for this reality is that the managers are very sensitive to the exclusive use of the managerial behaviors^(^ 10 ^)^ and that, during the 1980s and 1990s, many corporations reduced the number of intermediate managers in their organizational structures, thus extending the control scope for which several managers are responsible^(^ 10 ^)^.

On the other hand, there is low, positive, and significant concordance between both evaluations globally, according to the job position they held at the managerial level (Nursing Supervisors, Service Chiefs, and Shift Managers), and 19.3% with their subordinates. This finding is consistent with a study conducted in the United States of America, which signals that there is concordance as the professionals devoted to the development of Human Resources seek to educate and train their leaders on how to be more effective. In addition, as there is adjustment between the leadership behaviors the subordinates need and those they receive, there is a greater positive impact on the work performed, greater cognitive and affective confidence in the leader, and higher levels of favorable work in the employee^(^ 10 ^)^. Consequently, and due to the aforementioned, the institution can be benefited, since the necessary tasks to fulfill its mission will present more probability of success if there is concordance in the perceptions between the leaders self-evaluation and the evaluation by their subordinates.

There is evidence signaling that, as the congruence of the managers perceptions on their own competences increases, self-development needs decrease with time; which might induce us to think that, when employing appropriate feedback measures^(^ 26 ^)^, increasing self-knowledge as well as the knowledge of the subordinates, of the task to be performed, of the institution, and of the general setting, the concordance level might improve^(^ 22 ^)^.

The non-concordance detected in the managerial staff (80.7% of the population), is justified as an obvious indicator according to another study^(^ 13 ^)^ for two reasons, namely: first, the solitude of the leader might influence the way of evaluating and trusting the subordinates, which would have an additional impact on the role the leaders assign to their subordinates; and second: the solitude of the subordinates might influence how they judge their own skills, which would further affect their willingness to accept the roles that the leaders assign to them. It is for this reason that, according to an analysis of their job position, as the managerial level increases, so does the gap between self-esteem and the qualifications of others^(^ 26 ^)^.

The discrepancies between the findings can be the result, at least in part, of the instrument employed, of the characteristics of the population, of the methodology used, of diverse incentives, and of safeguarding against negative consequences when giving honest evaluations^(^ 26 ^)^ by the subordinates, elements that must be mitigated and considered in future studies.

What matters here is the contribution that could be made from the results obtained with the SLT and from the contributions of the communicational model, Joharis window^(^ 12 ^)^,since low concordance is situated in the ideal window, the free area, that is, both the leader and the subordinate that coincide with the leadership styles evaluated are in this area. Working on interpersonal learning will expand the free area and will reduce the other areas of this model^(^ 12 ^)^; for this reason, the institution will have to generate important changes, work on the level of trust between the two groups with criteria of giving and receiving feedback, strengthening assertive communication; that will ease the exchange of knowledge, conducts and expectations, aligned with the institutional vision, with facilitating work with their subordinates, with employing higher leadership styles and with greater variability, due to the use of the groups managerial skills.

The presence of such low concordance indicates that most of the leaders behavior is liberated and open to the subordinates and to other professionals; consequently, the tendency is lower for the collaborators to misinterpret or project wrong personal and work meanings on the leaders behavior^(^ 12 ^)^. The blind area between both groups must be avoided, since it hinders the improvement of the necessary interpersonal relationships to attain the higher leadership styles set forth by this situational theory; for this reason, the leaders must expand the free area and broaden its action radio together with their subordinates.

To the extent that access to information is enabled, the subordinates will feel more capable and with more power to make good decisions, congruent with the goals, objectives, and values of the organization^(^ 25 ^)^, since obstacle-free sharing and easing of information and communication motivates and generates confidence in the individuals to feel ownership towards the organization where they work. Direct and obstacle-free communication between the leader and the subordinates is the fundamental component of the organization; among other aspects, it reduces the danger of division among work peers, favors dialog, and maintains health, agility, flexibility, and fluency in the organization^(^ 25 ^)^. Thus, the subordinates can be able to innovate processes and to propose solution options to the leader, since the free area has been strengthened.

In view of the aforementioned, it is clear that efficient leaders are those who are willing to share their opinions on how to direct and motivate people with their subordinates^(^ 25 ^)^, but it is also evident that, to be an efficient leader, the individual must also have a sound point of view on leadership.

This study is very useful as a starting point, since it is necessary to further deepen the analysis through other methodological tools, given that it is very probable that both the leaders and the subordinates have biased their answers.

It is recommended that, in future research studies, the competences and the commitment of both leaders and subordinates are valued, as well as their maturity (level of preparation), in a personalized manner with objective methodologies that reduce the risk of bias in the answers. On the other hand, the limitations were the following: 1) small sample, 2) limited time for the staff to answer the surveys (both subordinates and leaders), 3) fear to express the desired answers in the instrument, 4) some subordinates had two leaders, and 5) limited theoretical and empirical studies that evaluated the situational leadership styles in the Mexican reality, conducting the analysis with Joharis window.

## Conclusion

It is necessary to work on the strengthening of the interpersonal relationships between the leader and the subordinates, as well as among peers, in order to attain assertive communication, strengthening trust, self-confidence, and the development of managerial skills and better leadership styles. Reason being that no single leadership style was found, but a great variety, as well as weak concordance.

The nursing managers employ different leadership styles, their predominance is task-oriented, it is necessary that there is a tendency towards superior styles targeted to the relationships, where the leaders and the subordinates development is favored in the different managerial skills, in the autonomous performance of the activities and with the use of motivation, factors which will favor empowerment in the care process and, consequently, its adequate management.

The subordinates do not perceive any notoriously prevailing single leadership style; they mainly identify Guiding, Directing and Participating, as well as Delegating to a noticeable lesser extent; in the case of the leaders, no single style is either perceived which is well-differentiated from the others. In other words, various styles have been found transitorily. It is because of this situation that it is likely that the subordinates potentialities are not exploited. On the other hand, the leaders must self-perceive that their subordinates recognize them as with communicative, teamwork, and motivation skills so that their self-knowledge, self-confidence, management ability, and strategic decision-making are enhanced.

The non-concordance between the Nurse in Chief and her subordinates can be due to multiple factors, namely: subjectivity, culture, work environment, and biases due to the interpersonal relationships both by the leader and by the subordinates.

The leaders must compel themselves to using the different tools offered by leadership, to being education promoters and examples for their subordinates, to expanding the spaces for feedback, communication, and interpersonal relationships; this is how we will advance from the leadership styles we have had for a long time and transcend the history of Nursing.

This type of study is useful to identify leadership and, with that, being able to potentiate the very managerial processes of the institution, increase the subordinates levels of motivation and creativity, and develop their abilities. Likewise, for future studies, it is necessary to add other variables like innovation and business performance in order to evaluate their relation with leadership concordance. Finally, this study contributes evidence of how situational leadership is being developed in a health setting, specifically in the Nursing staff. Additionally, no scientific evidence was found on the subject matter.
